# Secular trends and related factors in leisure-time sedentary behavior among Koreans: an analysis of data from the 2011-2017 Korea Community Health Survey

**DOI:** 10.4178/epih.e2022023

**Published:** 2022-02-14

**Authors:** Hyun-Ju Seo, Min-Jung Choi, Soon-Ki Ahn

**Affiliations:** 1 College of Nursing, Chungnam National University, Daejeon, Korea; 2 College of Nursing, The Catholic University of Korea, Seoul, Korea; 3 Department of Preventive Medicine, Chungnam National University Hospital, Daejeon, Korea

**Keywords:** Adults, Sedentary behavior, Socioeconomic factors, Health status, Public health

## Abstract

**OBJECTIVES:**

Sedentary behavior has gradually increased and has become a public health problem. Therefore, this study investigated trends in weekday leisure-time sedentary behaviors, as well as characteristic socio-demographic and lifestyle correlates in Korean adults.

**METHODS:**

We analyzed data from 914,946 adults aged ≥19 years who participated in the Korea Community Health Survey (2011, 2013, 2015, and 2017). Leisure-time sedentary behavior was categorized as a binary variable (<4 and ≥4 hr/day). Multivariable regression analysis was used to model the prevalence of sedentary behavior and estimate odds ratios.

**RESULTS:**

The prevalence of leisure-time sedentary behavior decreased from 15.2% to 14.4% in men and from 16.6% to 16.0% in women between 2011 and 2017, respectively. However, a significant increase was observed in subjects with an education lower than high school in both genders (β coefficient=0.12 for men and 0.08 for women, p for trend <0.001). Women in the lowest household income level (β coefficient=0.08, p for trend=0.001) and with poor subjective health status (β coefficient=0.05, p for trend=0.013) showed an increasing trend. Other factors associated with sedentary behavior were age, education level, body mass index, household income, walking activity, perceived stress level, and subjective health status in both genders.

**CONCLUSIONS:**

Identifying the secular trends and correlates of sedentary behavior by gender and associated factors will provide empirical evidence for developing public health campaigns and promotion programs to reduce sedentary behavior in Koreans.

## INTRODUCTION

Sedentary behavior is characterized by an energy expenditure of ≤ 1.5 metabolic equivalents (METs) of task while awake, in a sitting, reclining, or lying posture. Sedentary time is defined as the time spent in any context engaged in sedentary behavior. In the case of adults aged 18 years or older, the use of electronic devices such as a television, computer, or phone while sitting, reclining or lying down; reading, writing, or talking while sitting; and sitting in a bus, car, or train are all defined as sedentary behaviors [1,2]. Sedentary behavior has been an increasing public health problem, with a significant number of people reporting long durations of sedentary time. Americans spend 55% of their waking time, or 7.7 hr/day, in a sedentary state. Europeans were estimated to spend an average of 40% of their leisure time watching television (equal to 2.8 hr/day in the United Kingdom), a rate that is stable or increasing [3].

According to the Korean health statistics for 2018, adults in Korea aged ≥ 19 years reported an average of 8.3 hours of sedentary time per day. Only 8.9% of the adult population engaged in < 4 hours of sedentary time, while 20.6% of adults spent > 12 hours engaged in sedentary behaviors [4].

A sedentary lifestyle has been consistently reported to increase all-cause mortality as well as the risk of cardiovascular disease, diabetes, hypertension, and cancer [3,5]. Previous studies on increased leisure-time sedentary behavior reported increased all-cause mortality [6], decreased survival time [7], and a higher risk of cancer overall in women [8]. Thus, sedentary behavior, regardless of total sitting time or leisure-time sitting, has a substantial impact on mortality, comparable to those of tobacco use and obesity [9].

Although sedentary time is regarded as a public health disease and the importance of primary prevention has been emphasized [10], the time spent in sedentary behavior by adults in the United States significantly increased between 2001 and 2016 [11,12]. Additionally, the prevalence of sedentary behavior (> 4.5 hours of total sitting time/day) increased between 2002 and 2017 from 49.3% to 54.3% in adults in the European Union [13]. Previous studies on sedentary time and sedentary behavior conducted in Korea used data obtained from a specified period to investigate factors related to sedentary behavior [14,15]. No studies have analyzed secular trends according to gender, age, and related factors using community health survey data. Therefore, this study aimed to examine the temporal trends of weekday leisure-time sedentary behavior according to socioeconomic characteristics and to identify related factors affecting sedentary behavior.

## MATERIALS AND METHODS

### Participants and data collection

This study used a cross-sectional data series from the Korea Community Health Survey (KCHS), an ongoing, cross-sectional, nationally representative survey of the non-institutionalized Korean population using a stratified, multistage probability design. Since 2008, the KCHS has been a leading nationwide survey assessing the health and lifestyle behaviors of community-dwelling adults and the elderly. The survey used questionnaires to collect data on demographics, socioeconomic status, health conditions, and health-related behaviors. Trained interviewers completed the questionnaires while conducting household interviews [16]. The KCHS is administered by the Korea Disease Control and Prevention Agency (KDCA, formerly the Korea Centers for Disease Control and Prevention).

We used KCHS data obtained over consecutive 2-year cycles from 2011 to 2017. The same questionnaire was used to assess time spent on leisure during this period. Information on socio-demographic characteristics, lifestyle, and sedentary behavior was combined into a single dataset for each cycle from 2011 to 2017. Our study population consisted of adults aged 19 years or older. Individuals without complete data on sedentary behavior were excluded from the sample. There were 914,946 individuals included in the final sample.

### Assessment of sedentary behaviors

The question related to sedentary behavior was “How many hours a day did you spend sitting and, for example, watching TV, playing games, or using the Internet during your leisure time this past week (from Monday to Friday)?” The response options were as follows: less than 1 hr/day, 1-2 hr/day, 2-3 hr/day, and 4 hr/day or more. The total sedentary time in hr/day was used as a binary variable (< 4 and ≥ 4 hr/day) for the primary analyses. This cut-off value was used in a previous study in which hazard ratios for mortality and cardiovascular disease death significantly increased at 4 hr/day compared to less than 4 hr/day [17,18].

### Assessment of socio-demographic and lifestyle characteristics

Socio-demographic factors (i.e., age, gender, education level, body mass index [BMI], and average monthly household income) and lifestyle characteristics (i.e., average daily sleep time and walking activity per week) were investigated as factors related to sedentary behavior in previous studies [11,14,15,17]. Additionally, psychological factors (i.e., subjective stress level and subjective health status level) were selected as independent variables [19]. Participants were categorized into 3 age groups: 19-39 years old (young adults), 40-64 years old (middle-aged adults), and 65 years or older (elderly adults). Education level was categorized as less than high school, high school, or college and above [11]. BMIs were grouped according to the Asia-Pacific BMI classification [20]. There were 4 categories of average monthly household income (in US dollars [USD]): < 846, 846-2,536, 2,537-4,227, and ≥ 4,227 [19]. Average daily sleep time was divided into a dichotomous variable: recommended sleep duration for adults (i.e., 7-9 hr/day) and “other” [21]. Walking activity was categorized as a binary variable: engaged in the recommended amount of walking activity (i.e., walking for 30 minutes a day for 5 days or more per week) and “other” [22]. Self-rated levels of health were entered as “good” for the responses “very good,” “good,” or “moderate” and entered as “poor” for the responses “bad” or “very bad” [23].

### Statistical analysis

Survey analysis procedures were used to account for sample weights, stratification, and clustering of the complex sampling design to ensure nationally representative estimates [16]. Using the cross-sectional weights proposed by the KDCA, data from 4 cross-sectional surveys were collected every 2 years from 2011 to 2017, and a single data file was created to explore secular trends.

Estimates of crude weighted prevalence and 95% confidence intervals (CIs) of leisure sitting time (≥ 4 hr/day) were calculated by cycle, gender, and age group. Crude linear trends in sedentary behaviors were evaluated using linear regression models across survey cycles to estimate regression coefficients (β) and 95% CIs for every 2-year cycle. The p-value for the trends was estimated using the survey cycle as a continuous variable.

Logistic regression analysis was used to model the prevalence of sedentary behaviors and estimate odds ratios (ORs). Logistic regression analyses for secular trends during the entire period were conducted, using each year as an explanatory variable and sedentary behavior as a dependent variable (contrast vector value: 2011=-0.67082, 2013=-0.22361, 2015=0.22361, 2017=0.67082) [23]. Socio-demographic and lifestyle correlates for sedentary behaviors over time were identified using multivariable regression models adjusted for age, gender, education level, marital status, BMI, monthly household income, average daily sleep time, walking activity, perceived stress, and subjective health status level for all participants.

All statistical tests were 2-sided and statistical significance was set at p<0.05. The p-values were not adjusted for multiple tests and should be interpreted as exploratory. All statistical analyses were conducted using the R version 3.6.1 (R Foundation for Statistical Computing, Vienna, Austria).

### Ethics statement

The KCHS was approved by the Institutional Review Board of the KDCA. The raw KCHS data were downloaded from the KCHS website after applying for and receiving permission from the administrator of the KCHS website (May 2021). This study was approved by the Institutional Review Board of Chungnam National University (No. 202105-SB-076-01).

## RESULTS

### Subject characteristics

We included 914,946 participants aged ≥ 19 years from the 2011-2017 KCHS. The sample size per cycle ranged from 228,381 to 229,226 participants. The unweighted and weighted sample sizes in the 2011-2017 cycle overall and for each age group, according to socio-demographic and lifestyle characteristics, are presented in Table 1. In the elderly adult category (≥ 65 years), 24.9% of participants were men and 29.3% were women; women were generally older than men. Compared with women, men were more likely to be obese and more commonly had education levels of college or above (Table 1). Participants with missing covariates (10.1%) were excluded from the multivariate analyses.

### Trends in the prevalence of sedentary behavior

From 2011 to 2017, the prevalence of sedentary behavior decreased from 15.18% to 14.40% in men and from 16.62% to 16.01% in women. The secular trend in sedentary behavior showed a statistically significant decrease in both genders (β coefficient=-0.06 for men, -0.04 for women, p for trend <0.05). In men, the estimated prevalence of sitting time (≥ 4 hr/day) showed a statistically significant decline across all age groups (β coefficient=-0.09 for young adults; -0.11 for middle-aged adults; -0.05 for elderly adults, p for trend < 0.05). While the time spent engaged in sedentary behavior by elderly adult women increased over time, this was not statistically significant (β coefficient=0.01, p for trend > 0.05) (Table 2).

The prevalence of sedentary behavior in both gender significantly decreased over time among those with a college or higher level of education, a normal BMI, the highest household income level, appropriate average daily sleep time, the recommended amount of walking activity, some level of perceived stress, and good self-rated health status. In contrast, a significant increase in sedentary behavior was observed in those with an education level less than high school in both genders (β coefficient=0.12, p for trend < 0.001 for men; ß coefficient=0.08 for women, p for trend < 0.001). Women with the lowest household income level and poor self-rated health status also showed an increasing trend of sedentary behavior over time (β coefficient=0.08, p for trend=0.001 for household income level; β coefficient=0.05, p for trend=0.013 for poor self-rated health status) (Table 2).

### Factors related to sedentary behavior

The factors associated with sedentary behavior in both genders were age, education level, BMI, average monthly household income, walking activity, perceived stress level, and subjective health status. Participants who reported a higher estimated prevalence of sedentary behavior were elderly individuals of both genders, had less than a high school education, had a higher BMI, were from families with lower household income, did not engage in the recommended level of walking, and had a negative response to the subjective health status question (Table 3).

Additionally, men with the lowest household income level and poor self-rated health status were highly likely to exhibit sedentary behavior (adjusted odds ratio [aOR], 2.02; 95% CI, 1.92 to 2.12 for those with a household income level < 846 USD/mo and aOR, 2.03; 95% CI, 1.96 to 2.09 for poor self-rated health status). For women, the aOR in the obese group was 1.44 (95% CI, 1.40 to 1.48) compared with the normal-weight group; for those with the lowest household income, the aOR was 1.47 (95% CI, 1.40 to 1.53) compared with the highest household income group; and for those with a poor self-rated health status, the aOR was 1.59 (95% CI, 1.54 to 1.63) compared with the good self-rated health status group (Table 3).

## DISCUSSION

This study examined secular trends in leisure-time sedentary behavior using adult KCHS data. The estimated prevalence of leisure-time sedentary behavior > 4 hr/day generally decreased from 2011 to 2017. This decrease in the prevalence rate was statistically significant in men, women aged 19-64 years, and people with a high socio-demographic status and healthy lifestyle characteristics. However, leisure-time sedentary behavior showed an increasing tendency in women aged 65 years or older, those with less than a high school education, women with the lowest incomes, and those with negative subjective health status.

This study also aimed to identify gender-specific trends in leisure-time sedentary behavior in Korean adults from 2011 to 2017. Previous studies have presented inconsistent trends due to differing cut-off values for energy expenditure or time, and the use of varying longitudinal periods and covariates. Leisure-time sedentary behavior trends in Australian adults in 1992, 1997, and 2006 remained stable. This study reported that leisure-time computer use showed a significant increase, while other behaviors, such as reading, hobbies, and crafts showed slight reductions [24]. Among adults in the United States, the time spent watching television or videos remained stable at 2 hr/day or more; however, computer use during leisure time of 1 hr/day or more significantly increased from 2003 through 2016 [12]. In Dutch adults, leisure-time sedentary behavior remained relatively constant at about 60%, while leisure screen time increased by 26% between 1975 and 2005 [25]. However, that same study found that the percentage of overall leisure-time sedentary behavior did not increase due to an increase in occupational time of 4.7 hr/wk during the same period. Although a decrease of > 4 hr/wk leisure-time sedentary behavior was seen in our study, this finding provided a limited understanding of sedentary behavior as a whole, since the important variables of occupational time and occupational intensity [26] were not available in the KCHS. Therefore, additional standardized international research is required to accurately reflect trends that include important variables and to investigate cross-sectional surveys for the prevalence of leisure-time sedentary behavior.

In this study, leisure-time sedentary behavior was slightly increased in women > 65 years. This finding is consistent with a previously observed trend in adults in the United States from 2003-2016 [12] which defined sedentary behavior as watching television for more than 2 hr/day and using a computer over 1 hr/day. Older adults with high levels of leisure-time sedentary behavior were associated with low physical functionality, after adjusting for confounding variables [27]. In a systematic review, nearly 60% of older adults reported sitting for more than 4 hr/day, 15% of whom reported watching television for more than 4 hr/day during that period [28]. These findings indicate that older adults have more leisure-time sedentary behavior than is recommended by public health policies. Therefore, reducing sedentary time is important for older adults. Considering that this study found no significant decreasing trend in leisure-time sedentary behavior for women aged ≥ 65 years, a detailed health promotion campaign is necessary to change the socio-cultural environment and reduce the overall sedentary time of older adults.

In agreement with previous studies, this study identified that trends of prolonged leisure-time sedentary behavior were associated with low socioeconomic factors [26,29,30]. The trends in leisure-time sedentary behavior showed no significant change among individuals with lower than college education and those with low incomes. More leisure screen time, such as watching television, was correlated with lower formal education and lower income [12,31,32]. In addition, income directly influenced leisure-time physical activity and sedentary behavior [33]. Considering the trends of overall leisure-time sedentary behavior among individuals with low socioeconomic status, prioritized and focused interventions designed to decrease leisure-time sedentary behavior and reduce disparities are necessary from a public health perspective.

In this study, the trends of leisure-time sedentary behavior decreased in both men and women. However, the prevalence in women was consistently higher than in men between 2011 and 2017. Systematic reviews on sedentary behavior by gender revealed inconsistent results [31,34,35]. A previous study using accelerometer-measured sedentary times reported that women had higher levels of sedentary time than men from 2007 to 2009, but there were no significant differences according to gender from 2007 to 2017 [32]. Leisure-time sedentary behaviors among men typically involved computers and television, whereas women spent more time on reading and communication-based sedentary behaviors than men [35,36]. There was an increasing trend of leisure-time sedentary behavior in women with low education levels, low household incomes, and negative subjective health status (p<0.05). A previous study reported that increased sedentary time had a negative impact on subjective health status in women and stress recognition in both men and women [37]. Opportunities to decrease leisure-time sedentary behavior in women can also contribute to decreasing gender discrimination, lowering the gap in subjective health status differences, and lead to economic participation [38]. Further investigation of “gendered social and economic barriers,” [36] which influence leisure-time sedentary behavior, is necessary. It is also essential to develop public health programs that help to reduce sedentary behavior and that promote further studies to identify additional causes of sedentary behavior, particularly in relation to gender [32].

This study had several limitations. Self-reported leisure-time sedentary behavior may not reflect the true amount of sedentary time as compared to that measured with a device, such as a smartphone or accelerometer. Nevertheless, self-reported sitting time has been widely used in epidemiological studies [15] and measurement errors are unlikely to affect the secular trends over time. Second, it was not possible to evaluate contemporary trends in leisure-time sedentary behavior because the amount of sedentary leisure-time has not been investigated in the KCHS since 2019. Thirdly, although the typical variables related to sedentary behavior were included, other potentially important factors such as intensity of work-related time, depression, cognitive ability, and activity limitations were not included. Therefore, future research should consider a wider range of variables, including psychological, physical, cognitive, and environmental factors, as well as activity levels at work. Fourth, this study focused on sitting leisure-time per day on weekdays because the KCHS does not collect data on total sitting time per day; therefore, the inability to analyze particular types of sedentary behavior, including television viewing and computer use, or the total sitting time per day restricted possible comparisons between the results of this study and those of other studies. Finally, this study used cross-sectional data, which limited the ability to identify causal relationships between the variables and sedentary behavior.

In conclusion, this study identified secular trends in sedentary behavior depending on gender. The secular trends of leisure-time sedentary behavior in adults for at least 4 hr/day decreased in both women and men in the 19-64 years age group. The prevalence of sedentary behavior in women was consistently higher than that in men. No decreasing trend was found in women aged 65 years and older or in those with a high level of perceived stress. Associated factors will provide empirical evidence for developing public health campaigns and health promotion programs to reduce leisure-time sedentary behavior in Koreans. Future research should systematically and thoroughly examine the physical, psychological, socioeconomic, and environmental causes of high levels of leisure-time sedentary behavior in the general population and implement programs that can effectively reduce leisure-time sedentary behavior.

## Figures and Tables

**Table 1.** Sample size for sedentary behavior in the Korean population by socio-demographic and lifestyle characteristics, using 2011-2017 Korea Community Health Survey data

Variables		Men (n=411,052)	Women (n=503,894)	Men, weighted	Women, weighted
Year					
	2011	103,017 (25.1)	126,209 (25.0)	19,755,024 (24.20)	20,149,808 (24.24)
	2013	102,722 (25.0)	126,059 (25.0)	20,204,229 (24.75)	20,577,678 (24.75)
	2015	102,829 (25.0)	125,729 (25.0)	20,605,423 (25.24)	20,949,235 (25.20)
	2017	102,484 (24.9)	125,897 (25.0)	21,083,080 (25.82)	21,450,487 (25.80)
Age (yr)					
	19-39 (young adults)	109,144 (26.5)	125,599 (24.9)	31,589,830 (38.69)	29,477,017 (35.46)
	40-64 (middle-aged adults)	199,436 (48.5)	230,827 (45.8)	38,843,755 (47.57)	38,814,294 (46.69)
	≥65 (elderly adults)	102,472 (24.9)	147,468 (29.3)	11,214,171 (13.73)	14,835,896 (17.85)
Education level					
	College or above	164,979 (40.2)	147,816 (29.4)	43,706,963 (53.62)	34,861,951 (42.01)
	High school	130,087 (31.7)	132,420 (26.3)	25,215,200 (30.93)	24,639,488 (29.69)
	Less than high school	115,396 (28.1)	222,914 (44.3)	12,596,990 (15.45)	23,478,865 (28.29)
Body mass index (kg/m^2^)					
	18.5-22.9 (normal)	149,730 (38.1)	231,578 (50.7)	29,483,080 (37.40)	42,185,414 (53.63)
	<18.5 (underweight)	115,60 (2.9)	34,275 (7.5)	1,895,963 (2.40)	6,618,443 (8.41)
	23.0-24.9 (overweight)	107,216 (27.3)	95,129 (20.8)	21,246,333 (26.95)	15,167,370 (19.28)
	≥25.0 (obese)	124,527 (31.7)	96,077 (21.0)	26,210,962 (33.25)	14,693,924 (18.68)
Household income level (US dollar/mo)					
	≥4,227	74,1 3(18.6)	84,212 (17.3)	19,900,699 (25.24)	19,383,599 (24.23)
	2,537-4,227	112,662 (28.3)	124,625 (25.6)	26,364,908 (33.44)	24,874,452 (31.09)
	846-2,536	144,950 (36.4)	165,350 (33.9)	25,486,977 (32.33)	25,478,305 (31.85)
	<846	66,341 (16.7)	112,911 (23.2)	7,081,287 (8.98)	10,264,918 (12.83)
Average daily sleep time (hr)					
	7-9	206,469 (50.2)	249,047 (49.5)	39,510,882 (48.41)	40,920,890 (49.26)
	Other	204,387 (49.7)	254,362 (50.5)	42,109,571 (51.59)	42,156,866 (50.74)
Walking activity					
	30 min/day & ≥5 day/wk	209,756 (51.1)	242,058 (48.1)	45,258,033 (55.47)	43,629,544 (52.51)
	Other	201,004 (48.9)	261,516 (51.9)	36,333,646 (44.53)	39,452,502 (47.49)
Perceived stress					
	None	92,609 (22.5)	104,998 (20.9)	14,434,126 (17.69)	14,360,396 (17.29)
	A little	217,226 (52.9)	266,803 (53.0)	44,739,252 (54.82)	45,892,979 (55.24)
	Much	87,682 (21.3)	114,364 (22.7)	19,374,273 (23.74)	19,797,270 (23.83)
	Very much	13,289 (3.2)	17,181 (3.4)	3,068,232 (3.76)	3,025,094 (3.64)
Subjective health status					
	Good	341,636 (83.1)	377,413 (74.9)	71,999,505 (88.19)	68,337,331 (82.21)
	Poor	69,367 (16.9)	126,445 (25.1)	9,638,240 (11.81)	14,784,706 (17.79)

Values are presented as number (%).

**Table 2.** Crude weighted trends of leisure-time sedentary behavior among the Korean population, using 2011-2017 Korea Community Health Survey data

Variables		Men						Women					
		2011	2013	2015	2017	β	P for trend	2011	2013	2015	2017	β	P for trend
Overall		15.18 (14.86, 15.51)	14.50 (14.18, 14.83)	13.6 8 (13.38, 13.98)	14.40 (14.09, 14.71)	-0.06	<0.001	16.62 (16.32, 16.93)	15.87 (15.57, 16.18)	15.14 (14.83, 15.45)	16.01 (15.71, 16.30)	-0.04	<0.001
Age (yr)													
	19-39 (young adults)	15.31 (14.77, 15.85)	13.84 (13.31, 14.37)	12.83 (12.33, 13.33)	13.98 (13.44, 14.51)	-0.09	<0.001	15.84 (15.34, 16.35)	14.08 (13.60, 14.56)	13.43 (12.93, 13.93)	14.71 (14.20, 15.22)	-0.08	0.001
	40-64 (middle aged adults)	11.43 (11.05, 11.81)	10.75 (10.38, 11.12)	10.24 (9.88, 10.61)	10.10 (9.74, 10.46)	-0.11	<0.001	13.29 (12.90, 13.68)	12.22 (11.85, 12.60)	11.40 (11.04, 11.77)	11.66 (11.30, 12.02)	-0.12	<0.001
	≥65 (elderly adults)	29.44 (28.55, 30.33)	30.05 (29.18, 30.93)	27.61 (26.81, 28.42)	28.69 (27.87, 29.50)	-0.05	0.020	27.88 (27.09, 28.66)	29.51 (28.73, 30.29)	27.97 (27.21, 28.73)	28.78 (28.06, 29.50)	0.01	0.541
Education level													
	College or above	14.09 (13.62, 14.55)	12.32 (11.90, 12.75)	11.33 (10.94, 11.72)	11.69 (11.30, 12.08)	-0.17	<0.001	13.81 (13.32, 14.29)	11.77 (11.35, 12.19)	11.14 (10.73, 11.56)	11.79 (11.40, 12.19)	-0.13	<0.001
	High school	13.40 (12.90, 13.89)	13.86 (13.37, 14.36)	13.41 (12.91, 13.91)	14.29 (13.78, 14.81)	0.04	0.056	14.59 (14.10, 15.09)	14.09 (13.60, 14.58)	13.79 (13.28, 14.29)	14.81 (14.29, 15.33)	0.01	0.777
	Less than high school	21.85 (21.16, 22.54)	23.04 (22.31, 23.77)	22.95 (22.21, 23.69)	25.07 (24.28, 25.86)	0.12	<0.001	22.22 (21.67, 22.77)	23.47 (22.90, 24.04)	22.84 (22.26, 23.43)	24.54 (23.95, 25.13)	0.08	<0.001
Body mass index (kg/m^2^)													
	18.5-22.9 (normal)	15.74 (15.23, 16.25)	14.75 (14.26, 15.24)	13.99 (13.50, 14.47)	14.57 (14.06, 15.09)	-0.08	0.001	14.26 (13.87, 14.65)	13.43 (13.05, 13.81)	12.66 (12.27, 13.05)	13.57 (13.19, 13.96)	-0.05	0.002
	<18.5 (underweight)	26.16 (23.91, 28.42)	24.48 (22.34, 26.61)	26.14 (23.76, 28.53)	28.01 (25.60, 30.43)	0.08	0.177	16.60 (15.62, 17.58)	14.84 (13.83, 15.84)	13.70 (12.76, 14.65)	14.86 (13.81, 15.91)	-0.12	0.005
	23.0-24.9 (overweight)	13.27 (12.73, 13.82)	12.59 (12.07, 13.12)	12.17 (11.64, 12.69)	13.24 (12.70, 13.78)	-0.01	0.723	16.93 (16.27, 17.59)	16.01 (15.38, 16.65)	15.57 (14.93, 16.22)	16.06 (15.44, 16.68)	-0.05	0.044
	≥25.0 (obese)	14.95 (14.39, 15.50)	14.63 (14.07, 15.19)	13.52 (13.02, 14.02)	13.95 (13.46, 14.44)	-0.07	0.001	21.45 (20.70, 22.21)	20.79 (20.05, 21.54)	20.44 (19.73, 21.16)	21.16 (20.46, 21.85)	-0.01	0.534
Household income level (US dollar/mo)													
	≥4,227	11.42 (10.79, 12.06)	10.41 (9.88, 10.94)	9.51 (8.95, 10.08)	9.85 (9.32, 10.37)	-0.13	0.001	12.50 (11.88, 13.13)	11.68 (11.16, 12.20)	11.01 (10.41, 11.60)	10.87 (10.36, 11.39)	-0.12	<0.001
	2,537-4,227	12.20 (11.67, 12.73)	11.34 (10.84, 11.83)	10.59 (10.13, 11.04)	11.29 (10.81, 11.76)	-0.07	0.003	13.55 (13.03, 14.07)	12.89 (12.39, 13.39)	11.85 (11.38, 12.32)	13.18 (12.69, 13.67)	-0.04	0.102
	846-2,536	16.29 (15.76, 16.82)	16.68 (16.12, 17.23)	15.02 (14.51, 15.53)	17.05 (16.48, 17.62)	0.01	0.711	18.62 (18.08, 19.15)	18.05 (17.53, 18.58)	16.80 (16.27, 17.33)	18.08 (17.55, 18.61)	-0.05	0.014
	<846	28.90 (27.80, 30.00)	31.93 (30.76, 33.10)	29.01 (28.01, 30.02)	31.41 (30.33, 32.50)	0.05	0.067	25.68 (24.84, 26.53)	26.94 (26.08, 27.80)	25.01 (24.22, 25.81)	28.64 (27.80, 29.48)	0.08	0.001
Average daily sleep time (hr)													
	7-9	15.23 (14.78, 15.67)	14.67 (14.24, 15.11)	13.82 (13.39, 14.25)	14.42 (13.99, 14.85)	-0.06	0.001	15.83 (15.43, 16.23)	15.04 (14.65, 15.44)	14.10 (13.70, 14.50)	14.9 5(14.54, 15.35)	-0.06	0.001
	Other	15.12 (14.68, 15.56)	14.34 (13.91, 14.77)	13.55 (13.15, 13.95)	14.35 (13.94, 14.77)	-0.05	0.002	17.42 (16.99, 17.85)	16.66 (16.25, 17.08)	16.11 (15.70, 16.51)	16.97 (16.56, 17.37)	-0.03	0.057
Walking													
	Walking 30 min/day & ≥5 day/wk	14.57 (14.15, 15.00)	13.30 (12.90, 13.70)	12.93 (12.54, 13.32)	13.05 (12.66, 13.45)	-0.09	<0.001	14.68 (14.28, 15.08)	13.81 (13.43, 14.19)	13.42 (13.04, 13.81)	13.92 (13.54, 14.30)	-0.05	0.003
	Other	15.95 (15.48, 16.41)	15.95 (15.47, 16.42)	14.62 (14.18, 15.05)	16.16 (15.69, 16.63)	-0.01	0.509	18.80 (18.37, 19.24)	18.09 (17.66, 18.53)	17.07 (16.63, 17.51)	18.29 (17.87, 18.72)	-0.04	0.013
Perceived stress													
	None	20.07 (19.31, 20.83)	18.91 (18.16, 19.65)	18.52 (17.81, 19.23)	19.79 (19.06, 20.53)	-0.01	0.600	19.95 (19.24, 20.66)	20.11 (19.40, 20.82)	19.74 (19.03, 20.44)	20.56 (19.88, 21.25)	0.02	0.332
	A little	13.13 (12.73, 13.54)	12.60 (12.21, 12.98)	11.69 (11.31, 12.07)	12.24 (11.86, 12.62)	-0.07	0.001	14.47 (14.09, 14.85)	13.68 (13.32, 14.05)	12.89 (12.52, 13.25)	13.90 (13.54, 14.26)	-0.05	0.004
	Much	15.44 (14.81, 16.06)	14.72 (14.09, 15.35)	13.52 (12.94, 14.09)	14.35 (13.72, 14.97)	-0.08	0.001	18.19 (17.59, 18.80)	17.05 (16.47, 17.63)	15.95 (15.35, 16.54)	16.59 (15.97, 17.21)	-0.09	<0.001
	Very much	21.07 (19.28, 22.87)	20.56 (18.74, 22.38)	20.26 (18.59, 21.92)	19.68 (17.93, 21.42)	-0.06	0.267	22.91 (21.14, 24.68)	22.17 (20.50, 23.84)	21.30 (19.70, 22.90)	21.34 (19.70, 22.98)	-0.07	0.148
Subjective health status													
	Good	13.36 (13.03, 13.69)	12.49 (12.17, 12.82)	11.74 (11.44, 12.04)	12.24 (11.94, 12.55)	-0.08	<0.001	14.48 (14.16, 14.80)	13.48 (13.18, 13.78)	12.65 (12.34, 12.96)	13.50 (13.20, 13.81)	-0.07	<0.001
	Poor	28.69 (27.70, 29.69)	29.91 (28.90, 30.92)	28.22 (27.25, 29.19)	30.08 (29.07, 31.10)	0.03	0.260	26.32 (25.57, 27.07)	26.84 (26.09, 27.60)	26.77 (26.00, 27.55)	27.79 (27.02, 28.55)	0.05	0.013

Values are presented as weighted % (95% confidence interval).

**Table 3.** Weighted logistic regression models^1^ of leisure-time sedentary behavior, adjusted for socio-demographic and lifestyle characteristics, using 2011-2017 Korea Community Health Survey data

Variables	Men	Women
Year (continuous)	0.94 (0.92, 0.97)	0.96 (0.93, 0.98)
Age (yr)		
19-39 (young adults)	1.00 (reference)	1.00 (reference)
40-64 (middle aged adults)	0.65 (0.63, 0.67)	0.62 (0.60, 0.64)
≥65 (elderly adults)	1.40 (1.34, 1.46)	1.15 (1.10, 1.21)
Education level		
College or above	1.00 (reference)	1.00 (reference)
High school	1.03 (1.00, 1.06)	1.24 (1.20, 1.28)
Less than high school	1.14 (1.10, 1.19)	1.37 (1.31, 1.42)
Body mass index (kg/m^2^)		
18.5-22.9 (normal)	1.00 (reference)	1.00 (reference)
<18.5 (underweight)	1.40 (1.30, 1.50)	1.05 (1.00, 1.10)
23.0-24.9 (overweight)	0.92 (0.89, 0.95)	1.14 (1.11, 1.17)
≥25.0 (obese)	1.07 (1.04, 1.10)	1.44 (1.40, 1.48)
Household income level (US dollar/mo)		
≥4,227	1.00 (reference)	1.00 (reference)
2,537-4,227	1.07 (1.03, 1.11)	1.06 (1.02, 1.10)
846-2,536	1.36 (1.31, 1.41)	1.34 (1.29, 1.39)
<846	2.02 (1.92, 2.12)	1.47 (1.40, 1.53)
Average daily sleep time (hr)		
7-9	1.00 (reference)	1.00 (reference)
Other	0.97 (0.94, 0.99)	1.03 (1.00, 1.05)
Walking activity		
Walking 30 min/day & ≥5 day/wk	1.00 (reference)	1.00 (reference)
Other	1.20 (1.17, 1.23)	1.31 (1.28, 1.34)
Perceived stress		
None	1.00 (reference)	1.00 (reference)
A little	0.73 (0.70, 0.75)	0.75 (0.73, 0.78)
Much	0.83 (0.80, 0.86)	0.87 (0.84, 0.90)
Very much	1.10 (1.03, 1.17)	1.05 (0.99, 1.12)
Subjective health status		
Good	1.00 (reference)	1.00 (reference)
Poor	2.03 (1.96, 2.09)	1.59 (1.54, 1.63)

Values are presented as adjusted odds ratio (95% confidence interval).

1 Adjusted for survey year, age, education level, body mass index, household income level, average daily sleep time, walking activity, perceived stress, and subjective health status.
